# Callus Culture System from *Lonicera japonica Thunb* Anthers: Light Quality Effects on Callus Quality Evaluation

**DOI:** 10.3390/ijms26052351

**Published:** 2025-03-06

**Authors:** Jiali Cheng, Fengxia Guo, Wei Liang, Hongyan Wang, Yuan Chen, Pengbin Dong

**Affiliations:** State Key Laboratory of Aridland Crop Science, College of Agronomy, College of Life Science and Technology, Gansu Agricultural University, Lanzhou 730070, China; chengjiali24@icloud.com (J.C.); guofx@gsau.edu.cn (F.G.); liangw@gsau.edu.cn (W.L.); why8852@163.com (H.W.)

**Keywords:** *L. japonica*, callus culture, light quality, histomorphological structure, bioactive metabolite

## Abstract

*Lonicera japonica* Thunb has significant edible and medicinal value, possessing heat clearing, detoxification, antibacterial, and blood pressure reduction properties. Currently, its quality is constrained by factors such as climate, environment, flowering period, and germplasm degradation. The strategy of using bioreactors and abiotic inducers to produce bioactive metabolites has not yet been implemented. This study reports, for the first time, the induction of an embryogenic callus from *L. japonica* anthers, the identification of tissue morphological structures, and the effects of light induction on the callus morphology, metabolite accumulation, and antioxidant activity. The results showed that the MS medium, supplemented with 1.0 mg·L^−1^ 6-BA, 1.5 mg·L^−1^ NAA, 1.5 mg·L^−1^ 2,4-D, and 0.2 mg·L^−1^ KT, induced 89% embryogenic callus formation. Uniform callus lines were obtained using 2.0 mg·L^−1^ 6-BA, 0.5 mg·L^−1^ NAA, and 0.2 mg·L^−1^ KT in each subcultivation. Embryogenic cells were observed to have closely arranged spherical protruding granules on their surface, along with visible nuclei and numerous starch grains. After 15 days of blue light induction, active metabolites and antioxidant activities peaked. This experimental system not only provides support for germplasm innovation but also indicates that abiotic inducers can be utilized as a means to achieve higher yields of metabolic products.

## 1. Introduction

*Lonicera japonica* flos is the dried flower bud of *Lonicera japonica* Thunb, which is also known as Jin Yin Hua and Japanese honeysuckle [1]. It is a member of the Caprifoliaceae family and is highly regarded as a traditional herb in East Asia [2]. With a history spanning millennia, *L. japonica* stands as a cornerstone of Traditional Chinese Medicine, renowned for its multifaceted therapeutic attributes, including antibacterial, anti-inflammatory, antioxidant, antiviral, and anticancer properties [3,4,5,6,7]. Its pharmacological activities primarily stem from secondary metabolites, including organic acids, flavonoids, iridoids, saponins, etc. [8,9]. For instance, phenolic compounds play significant roles in anti-inflammatory, anti-aging, and antioxidant activities, while flavonoids exhibit positive effects in treating chronic diseases such as coronary heart disease, cancer, and diabetes [10,11]. Furthermore, it has been employed as a dietary additive in tea and in other beverages, and it is known as a “Functional Food” [12].

According to “The Statistics from the National Economic Forest Association”, the global demand for *L. japonica* has surpassed 20 million kilograms, generating an output value of up to USD 860 million, positioning it as one of the most economically significant medicinal plants [13]. However, due to limited land resources, large-scale cultivation is unsustainable [14]. Furthermore, the prolonged cultivation period with limited varietal improvements has restricted both the quality and production of medicinal compounds [15]. Additionally, the production of secondary metabolites in field-grown plants is influenced by geographical, seasonal, and climatic factors [16]. Moreover, extracting and purifying these metabolites from whole plants is a labor-intensive and time-consuming process, while the bioactivity of the obtained compounds remains relatively low [17]. In order to prevent these problems from worsening further, it has been found that plant cell culture has the benefits of stability, sustainability, and being free from limitations. Utilizing plant cell cultures or callus cultures is an important condition for conducting biotechnology research and is also the basis for further realizing somatic embryogenesis or in vitro plant regeneration [18,19]. Moreover, inducing the production of plant secondary metabolites is a feasible and effective strategy [15]. During recent years, some natural bioactive substances, for instance, anthocyanins, ginsenosides, steviol glycosides, paclitaxel, and flavonoids, have been successfully studied and commercially used in plant cell cultures [16,19,20,21]. Therefore, the application of *L. japonica* cell culture for producing natural, bioactive compounds holds significant commercial potential and can fulfill the growing demand for these substances in the pharmaceutical, food, and cosmetic sectors.

Several strategies have been proposed for the synthesis of secondary metabolites in plant cell cultures. Among these, elicitors are considered one of the most effective strategies for enhancing secondary metabolite synthesis in plant cell cultures [22,23]. Among various biotic and abiotic elicitors, light is a key abiotic factor that plays a vital role in regulating the growth patterns of in vitro cultured tissues while enhancing the synthesis of secondary metabolites [24]. Previous studies have confirmed the enhancing effect of light on the accumulation of secondary metabolites [25,26,27]. For example, in callus cultures of *Stevia rebaudiana*, there were improvements observed in total phenolics, total flavonoids, and total antioxidant capacity [28]. In light-induced callus cultures of *Artemisia absinthium*, red light improved peroxidase activity, protease activity, total protein content, and the chlorophyll a/b ratio. Green light enhanced the accumulation of total phenolics, total flavonoids, and antioxidant activity. Yellow light increased the malondialdehyde (MDA) content, while both white and green light improved the total chlorophyll and carotenoid content [24]. Similarly, under induction by different light qualities, biomass growth and the synthesis of antioxidant secondary metabolites were markedly increased in *Prunella vulgaris* callus cultures [29]. Moreover, in red callus cultures of *Vitis vinifera*, morphological growth, phenolic compound accumulation, and total antioxidant capacity exhibited significant changes in response to different light qualities [30].

Callus tissues can be derived from different explants, with their characteristics differing based on their source. For instance, in *Cnidium officinale*, root explants demonstrate a higher callus formation rate than stem explants [31]. In *Centella asiatica* (L.), triterpenoid secondary metabolites are abundantly present in roots induced from leaf explants [32]. When callus tissues induced from leaves, stems, and roots of *Hypericum triquetrifolium Turra* were treated with gamma irradiation, it was found that the content of p-OH-benzoic acid was higher in callus tissues induced from leaf explants, while the highest content of chlorogenic acid was observed in callus tissues induced from root explants [33]. In *Lycium barbarum* L., among different explants (leaf, petiole, root, hypocotyl, and node), hypocotyl explants produced callus tissues with the largest diameter [34]. Anthers of black rice can produce light yellow and spherical sporogenous callus tissues [35]. While callus induction from various explants of *L. japonica* has been reported in previous studies [14], callus induction from anther explants of *L. japonica* has not been reported.

In this study, *L. japonica* anthers were used as the source material to develop a method for inducing callus tissues, resulting in the generation of high-quality callus cultures. Morphological and cytological analyses of the callus tissues revealed both common and distinct features. The establishment of a callus culture system from *L. japonica* anthers provides a foundation for the efficient production of homozygous plants and the enhancement of *L. japonica* germplasm. Furthermore, elicitor-mediated techniques play a key role in boosting secondary metabolite accumulation, enhancing the quality of medicinal plants, and opening new possibilities for pharmaceutical advancements.

This study used *L. japonica* anthers as material and successfully established a method for inducing callus from *L. japonica* anthers, obtaining high-quality callus cultures. Through morphological and cytological observations of the callus, the unique characteristics of embryogenic and non-embryogenic callus types were revealed. The established *L. japonica* anthers callus culture system provides strong support for the rapid improvement of *L. japonica* germplasm resources. Additionally, the application of inducer technology provides favorable conditions for promoting the accumulation of secondary metabolites.

## 2. Results and Discussion

### 2.1. Association Between Flower Bud/Floret Size and Developmental Stage of Microspores

**Figure 1:**
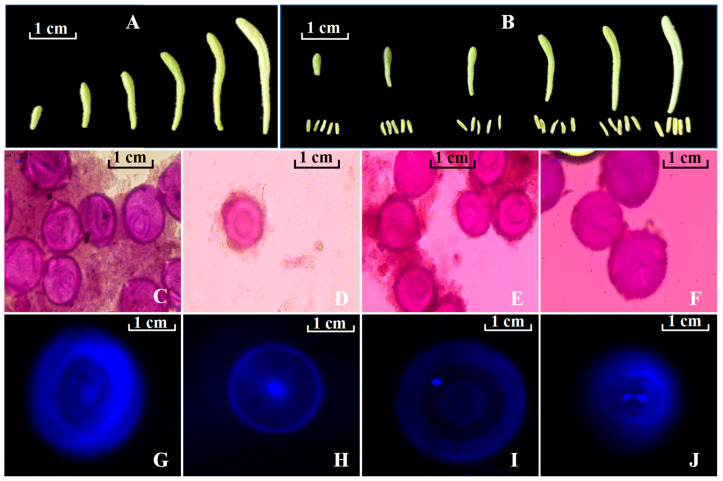
Different flower bud sizes correspond to distinct microspore developmental stages. (**A**,**B**) represent flower buds of different sizes; (**C**–**J**) depict the cytological features of the developmental stages of microspores in *L. japonica*. (**C**–**F**) were stained with Carnoy’s solution; (**G**–**J**) were stained with DAPI. (**C**,**G**): tetrad stage; (**D**,**H**): early uninucleate stage; (**E**,**I**): late-uninucleate stage; and (**F**,**J**): binucleate stage.

The size of floral buds determines the developmental stage of microspores, influencing the success rate of anther tissue culture [36,37]. The cytological observation of *L. japonica* floral buds revealed distinct developmental stages of microspores: tetrad, early uninucleate, late-uninucleate peripheral, and binucleate stages, corresponding to buds of different sizes (Figure 1A–J). Floral buds reaching 0.5–0.7 cm in length, displaying greenish coloration with short corollas, predominantly contain microspores at the tetrad stage (Figure 1C,G). When the buds grow to a length of 1.0–1.4 cm, showing a greenish hue and having elongated corollas, the majority of microspores progress to the early uninucleate phase (Figure 1D,H). Subsequently, at lengths of 1.8–2.6 cm (Figure 1E,I), accompanied by notable elongation of corollas and lightening of green coloration, most of the microspores were in the late-uninucleate stage. Finally, when buds reach a length of 2.8–3.8 cm, displaying a yellowish-green hue and corolla length triple that of the calyx, microspores are predominantly in the binucleate stage (Figure 1F,J). Research by Kumar and Alan has indicated that anther cultures containing microspores at the late-uninucleate peripheral stage yield the highest number of embryos, shoots, and haploids when cultured on MS basal medium [38,39]. In this experiment, the induction rate of the callus tissue was highest at 78% when microspores in the anthers were at the late-uninucleate peripheral stage, 6.5 times higher than that at the binucleate stage. Additionally, the browning rate was lowest at 15% during this stage, showing a significant difference compared to the other three stages (Table 1). Previous studies have reported lower induction rates when anthers at the binucleate stage are used for cell culture, which is attributed to the accumulation of starch and other storage material in the later stages of the microspore development, which often leads to a loss of embryonic potential [40,41]. These findings are consistent with our study results.

### 2.2. Callus Induction

The concentrations of plant growth regulators (PGRs) were optimized by the L_9_ (3^4^) orthogonal design to promote the callus induction and morphogenetic characteristics of *L. japonica*. The main PGRs employed included auxins (NAA, 2,4-D, IBA) and cytokinins (6-BA, KT), which have been extensively documented [42,43]. Iiyama et al.’s research has shown that different combinations of PGR concentrations significantly affect the callus induction rate and morphogenic characteristics [44]. This emphasizes the importance of appropriate PGR combinations in promoting callus formation and growth. The results of this study indicated that under different PGR combinations, the callus induction rate ranged from 9% to 81%, and significant differences in callus color were observed (Table 2). An analysis of variance (ANOVA) of the orthogonal experiments (Table 3) revealed that NAA was the primary hormone influencing callus formation and growth, followed by 6-BA, 2,4-D, and KT at significant levels (*p* < 0.05). However, the theoretical value of A_2_B_3_C_3_D_1_ in Table 3 was different from the A_2_B_3_C_1_D_2_ combination in Table 2. Consequently, a comparative analysis between A_2_B_3_C_1_D_2_ and A_2_B_3_C_3_D_1_ was conducted, revealing that the induction rate of A_2_B_3_C_3_D_1_ was 89%, with yellowish friable callus characteristics, establishing it as the optimal PGR combination. Furthermore, the results of this study show that higher concentrations of NAA promote the formation of callus tissues, consistent with the findings of Wu et al. and Rout et al. [45,46]. Additionally, studies have shown that yellow and loose callus tissues are rich in secondary metabolites, making them valuable and sustainable raw materials in commercial markets [47,48].

### 2.3. Callus Proliferation

After transferring 0.5 g of yellow and loose callus tissue onto a fresh culture medium identical to that used for induction, it was observed that the hormone ratios optimized for induction were not suitable for a subsequent subculture. Consequently, an optimization of plant growth regulator (PGR) combinations for the subculture was conducted based on an L_9_ (3^4^) orthogonal experiment. In Table S1, the A_2_B_3_C_1_D_2_ combination exhibited the highest fresh weight. Additionally, based on the Rk value analysis (Table S2), the priority ranking of PGRs affecting callus induction was B > A > C > D, aligning with the ANOVA results.

The A_2_B_3_C_1_D_2_ combination closely matched the theoretical values, validating its efficacy. Thus, the optimal PGR combination for subculture was identified as 2.0 mg·L^−1^ 6-BA, 0.5 mg·L^−1^ NAA, and 0.2 mg·L^−1^ KT. Compared to induction, the subculture requires elevated cytokinin concentrations to stimulate cell division and facilitate bud formation [49].

### 2.4. Histological Observations

**Figure 2:**
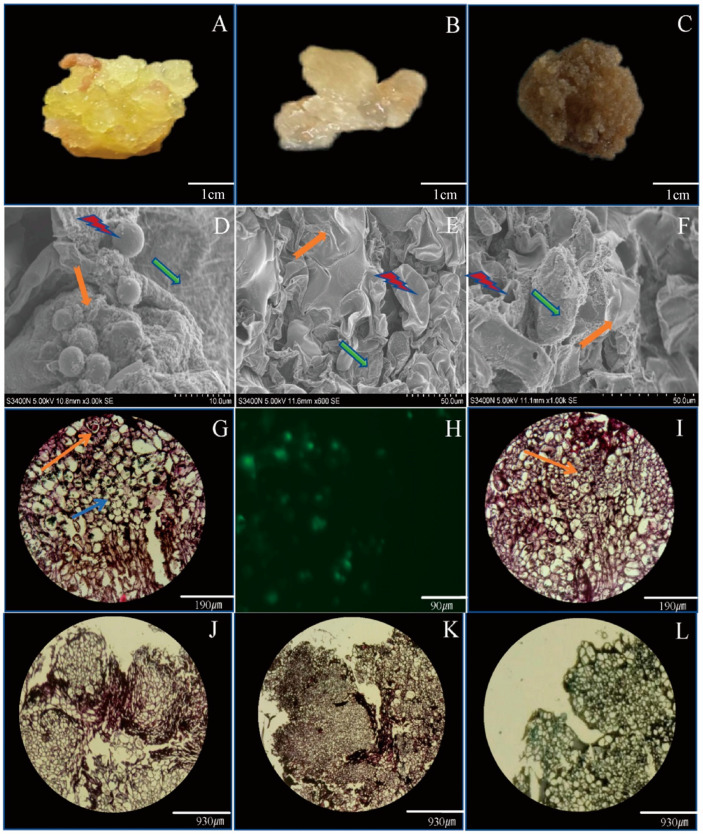
Morphological and histological observations of different callus tissues. (**A**) Yellow embryogenic callus, (**B**) light yellow non-embryogenic callus, and (**C**) brown necrotic callus. (**D**) Embryogenic callus surface, (**E**) non-embryogenic callus surface, and (**F**) necrotic callus surface. SEM images show spherical protrusions, depressions, ruptures (marked with red lightning bolts), granular structures (marked with orange thick arrows), and membrane layers (marked with green arrows). (**G**,**I**) Electron microscopy images of embryogenic and non-embryogenic tissues, respectively. Nucleus (marked with orange thin arrows) and starch granules (marked with blue arrows). (**H**) In the embryogenic callus, starch granules emit green fluorescence under dark field microscopy. (**J**–**L**) Embryos with spherical, heart-shaped, and cotyledon-like structures were observed in the embryogenic callus.

The identification of an embryogenic callus plays a vital role in ensuring a successful subculture in plant tissue culture [50]. The callus derived from explants displays irregular shapes, colors, and structures. Inducing a callus from *L. japonica* anthers yields different types of callus. The type I callus is yellow with a moist and dense surface (Figure 2A). The type II callus is light yellow, containing a high water content and a loose structure (Figure 2B). The type III callus is brown and the surface is dry and dense (Figure 2C). Scanning electron microscopy (SEM) shows that the surface of the callus has small pores, dense continuous layers, and granular structures (Figure 2D–F). The surface cells of the three callus types transition from tightly packed and spherical to indented, and finally to broken and loose cells. These structures facilitate the clear distinction among embryogenic, non-embryogenic, and browning callus. The current research findings are supported by Binte Mostafiz et al. [51]. Similarly, Corral-Martínez et al. reported that during embryogenesis, most angiosperms exhibit an embryogenic callus with distinct granular and fibrous surface structures [52]. Paraffin section observations reveal that the type I embryogenic callus exhibits a uniform cell size, active cell division, visible nuclei, and numerous starch grains (Figure 2G,H). Additionally, embryogenic cells were observed to develop from globular to cotyledonary embryos (Figure 2J–L). Starch grains stain green with safranin-fast green stain, while nuclei stain red. Under dark-field optical microscopy, starch grains exhibit spontaneous green fluorescence (Figure 2H). In contrast, the non-embryogenic callus (Type II and Type III) exhibits uneven cell sizes, a disorganized arrangement, fewer nuclei, and a notable lack of starch grains (Figure 2I). Many plants exhibit similar histological features when comparing embryogenic and non-embryogenic tissues. For instance, in Camellia, the embryogenic callus has a dense cytoplasm and well-defined nuclei, whereas the non-embryogenic callus has a less-dense cytoplasm and fewer nuclei [53]. A comparable pattern has also been documented in *Handeliodendron bodinieri* L. [54]. Starch grains are present in the embryogenic callus but absent in the non-embryogenic callus, a finding consistent with Kong et al.’s observations [55]. Thus, the presence or absence of starch grains provides a convenient tool to identify embryogenic and non-embryogenic tissues. In the regeneration system of Angelica, somatic embryos within the embryogenic callus were seen progressing from the globular to cotyledonary stages [56]. Similar observations were made in *Juglans regia* L. and *Pulsatilla tongkangensis* [57,58].

### 2.5. Influence of Different Light Qualities on the Growth and Development of L. japonica Callus

**Figure 3:**
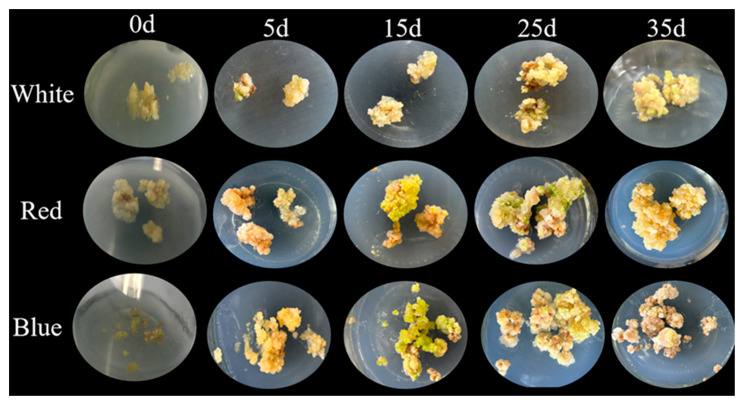
Effects of different light qualities on proliferation of *L. japonica* callus tissues.

This study observed surface cell color changes under various light qualities (Figure 3). After 15 days of cultivation, the callus exposed to red and blue light turned green. The blue light treatment not only changed the surface color to green but also caused the green color to spread internally. In contrast, exposure to red light resulted in a slower shift in color, with greening only on the surface, while white light induced no visible color change. By the 25th day, the callus continued to proliferate, and the green color deepened under red light but lightened under blue light. After 35 days, the multiplication of the callus under red and blue light slowed down with signs of senescence, and the surface color faded and lost its luster. In addition, more brown tissue and white sclerotia formed on the surface of the callus with the increase in incubation time. Senescence was most pronounced under blue light, where brown cell formation significantly increased. Similar results were reported by Lai et al., who observed the most severe callus senescence under blue light and the least under white light [30].

### 2.6. Effect of Light Qualities on the Accumulation of Total Phenols, Total Flavonoids, and Soluble Sugars

Phenolic compounds are widely found in plants and are appreciated for their antioxidant properties [59,60].

**Figure 4:**
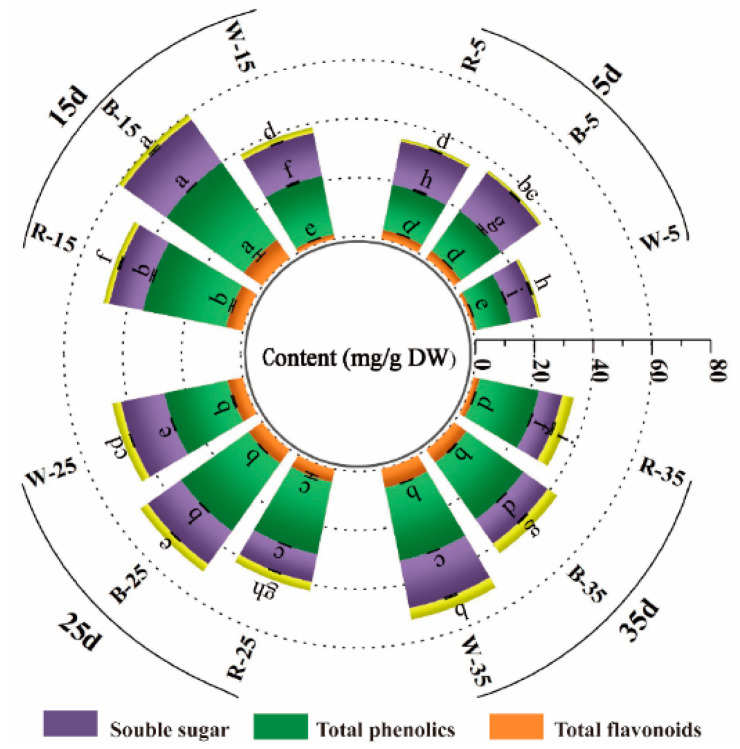
The impact of various light qualities on the accumulation of total flavonoids, total phenols, and soluble sugar in *L. japonica* callus tissues at 5, 15, 25, or 35 days. Different lower letters above the bar graphs indicate significant differences (*p* < 0.05).

Total flavonoids are a class of polyphenolic compounds present in plants. Soluble sugars are the basis of plant metabolism and an important source of energy required for growth and metabolism [61,62]. In this study, we investigated how light quality affects the accumulation of phenols, flavonoids, and soluble sugars in L. japonica callus with different induction durations (Figure 4).

The total phenol content of *L. japonica* callus differed significantly under different light qualities. In contrast to white light, both blue and red light significantly (*p* < 0.05) improved the total phenol content during the first three stages of *L. japonica* callus. The total phenol content varied from 16.71 to 32.40 mg g^−1^ DW under blue light irradiation; the range was 15.775–28.819 mg g^−1^ DW under red light irradiation. The phenolic content induced by blue light was significantly higher than that by red light. The highest total phenolic content of 32.398 mg g^−1^ DW was found in the healing tissues treated with blue light for 15 days. At 35 days of incubation, the total phenolic content was 25.408 mg g^−1^ DW in white light treatment, which was 0.069 and 0.23 times higher than that in blue light and red light irradiation, respectively.

The total flavonoid content in the callus exhibited significant variation with different light conditions. The total flavonoid content in the healing tissues showed significant differences with different light conditions. Under the white light condition, the total flavonoid content rose with the cultivation time and peaked at 6.42 mg g^−1^ DW at 35 days. It was not significantly different from that of the blue light treatment, but 2.01 times higher than the red light treatment (*p* < 0.05). The total flavonoid content increased and then decreased under blue light and red light conditions, respectively, reaching a peak at 15 days at 7.653 mg g^−1^ DW under blue light and 0.33 times higher under red light.

The light quality also had different effects on the soluble sugar content. Compared with white light, blue light remarkably facilitated the accumulation of soluble sugars at 5 and 15 days of incubation (*p* < 0.05). The soluble sugar content in the blue light reached a maximum value of 17.779 mg g^−1^ DW at 15 days, which was 0.20 and 0.51 times higher than that in the white and red light, respectively. However, blue light inhibited the accumulation of soluble sugars at the latter two stages of incubation. Red light promoted the accumulation of soluble sugars only at 5 days incubation, with a content of 14.868 mg g^−1^ DW. Under white light, the soluble sugar content increased with the increase in incubation time and reached a peak value of 16.443 mg g^−1^ DW at 35 days.

These results indicate that blue light induction for 15 days can significantly promote the accumulation of total phenols, flavonoids, and soluble sugars in *L. japonica* callus. A number of studies have demonstrated that blue light can induce the accumulation of total phenols, flavonoids, and soluble sugars [28,63,64,65]. Blue light was found to stimulate the expression of phenylalanine deaminase (PAL), a key enzyme for phenylpropane synthesis in the phenolic compound synthesis pathway [29,66]. Earlier studies have also revealed that blue light stimulates an increase in the total phenolic content and total flavonoid content in the healing tissues of *Rhodiola imbricata* and *Stevia rebaudiana* [28,63]. In addition, the accumulation of soluble sugars in non-bearing cabbage was also enhanced by LED blue light [64] and similarly promoted in *Hyoscyamus reticulatus* [65].

### 2.7. Effect of Light Quality on Antioxidant Capacity

DPPH and FRAP assays are widely used to assess the antioxidant capacity of extracts due to their simplicity and visibility [67,68].

**Figure 5:**
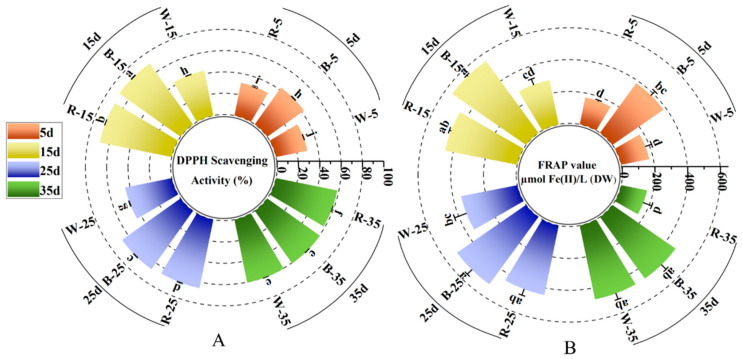
Effects of different light qualities on the DPPH and FRAP content in *L. japonica* callus at 5, 15, 25, and 35 days. (**A**) DPPH content in *L. japonica* callus. (**B**) FRAP content in *L. japonica* callus. Different lower letters above the bar graphs indicate significant differences (*p* < 0.05).

In this study, we used DPPH free radical scavenging activity to assess the antioxidant capacity of callus tissues under different light qualities (Figure 5A). Both blue and red light significantly promoted the scavenging activity of DPPH radicals in the first three periods of incubation compared with white light, and the DPPH activity was significantly higher under blue light than red light (*p* < 0.05). At 15 days of incubation, the DPPH scavenging rate of the blue light-treated group reached a maximum of 70.25%, which was higher than that of white light and red light by 63.4% and 2.15%, respectively (*p* < 0.05). Nevertheless, at 35 days of induction, red light significantly inhibited the DPPH radical scavenging activity, whereas the difference between blue light and white light was not significant.

Ferric reducing antioxidant capacity (FRAP) determination also showed remarkable differences (Figure 5B). In comparison with white light, both blue and red light significantly increased the antioxidant capacity of *L. japonica* callus in the first three stages of incubation (5, 15, and 25 days). Furthermore, at 15 days, blue light induced the highest value of antioxidant capacity, which was 0.96 times (*p* < 0.05) that of white light and 0.23 times (*p* > 0.05) that of red light. However, at 35 days, the FRAP value of the healed wounds induced by white light was 437.882 µmol Fe (II)/L DW, which was 1.92 times higher than that obtained by red light (*p* < 0.05), and there was no significant difference between the two groups and the blue light treatment group (*p* > 0.05). The results of our study are in agreement with Biswal et al., who reported a similar elevated antioxidant capacity of callus cultures of *Operculina turpethum* (L.) exposed to blue light [69]. Along the same lines, Azad et al. revealed higher DPPH and FRAP activities in soybeans treated with blue light [70]. Both of these findings emphasize the important role of blue light in enhancing the antioxidant capacity of callus cultures, which was reflected in the boost in DPPH and FRAP activities.

### 2.8. Chromatographic Fingerprint Analysis

**Figure 6:**
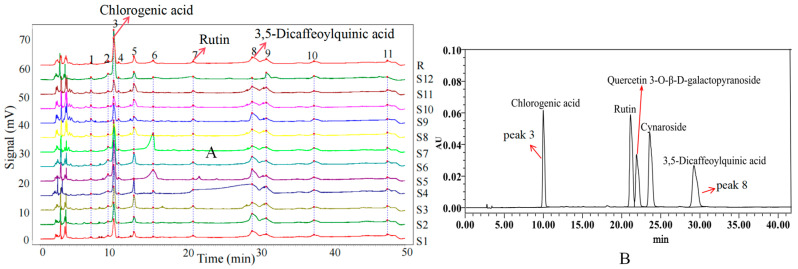
(**A**) The standard fingerprints of *L. japonica* in different light qualities. The right serial numbers (S1–12) represent blue: 5d, blue: 35d, white: 35d, red: 35d, red: 25d, blue: 25d, red: 15d, white: 15d, white: 25d, white: 5d red: 25d, blue: 15d; (**B**) reference fingerprint.

Chromatographic fingerprinting is a comprehensive method for the qualitative and quantitative analysis of a wide range of chemical components in Traditional Chinese Medicines. High-performance liquid chromatography (HPLC) remains the primary method for TCM quality control thanks to its good sensitivity, high separation, and high efficiency [71]. Using the established HPLC method, the fingerprint chromatogram of the S1 sample was imported into the “Traditional Chinese Medicine Chromatographic Fingerprint Similarity Evaluation System (2012 Edition)” software, where a reference chromatogram was set. By automatically matching common peaks and applying multipoint correction, a standard fingerprint chromatogram was subsequently generated [72]. The standard fingerprint profile of *L. japonica* callus tissue is shown in Figure 6A. Based on the chromatographic peak matching information, 11 common peaks were identified. The similarity analysis revealed that the similarity values between the samples with different light qualities treatments and the standard fingerprints ranged from 0.872 to 0.991, which indicated that the chemical compositions of the callus differed to a certain extent under different light conditions. The similarity values are shown in Table 4.

### 2.9. Principal Component Analysis

**Figure 7:**
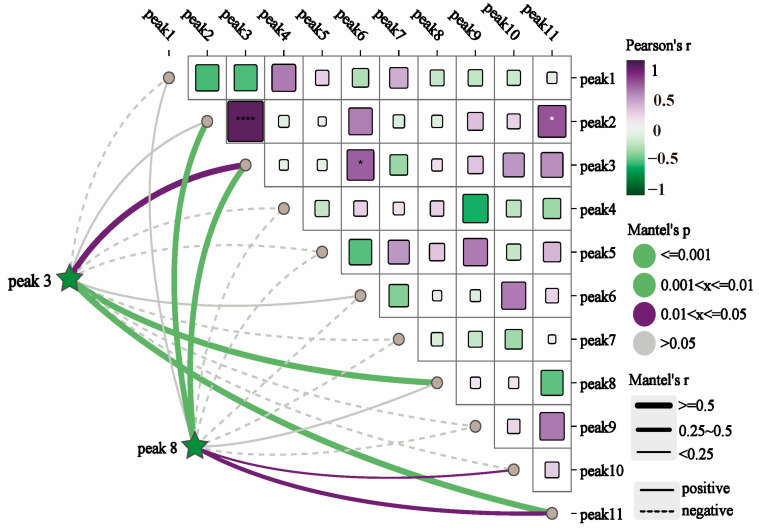
Correlation between common peak-to-peak areas.

**Figure 8:**
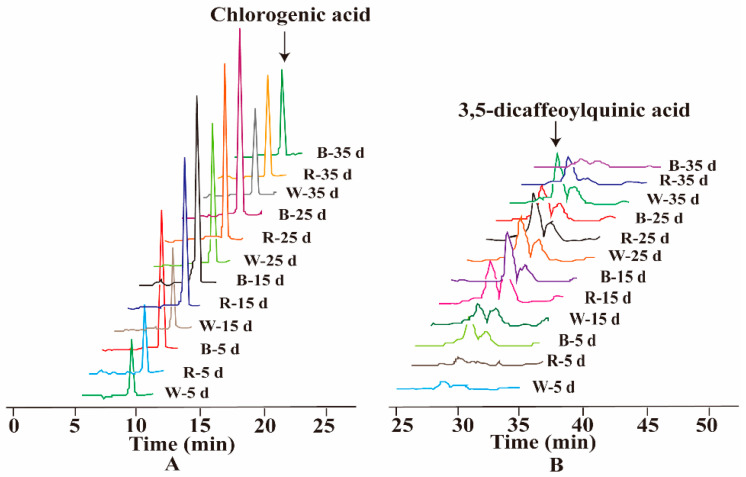
High-performance liquid chromatography of the effect of different light qualities on chlorogenic and 3,5-dicaffeoylquinic acids. (**A**) Chlorogenic acid chromatography. (**B**) 3,5-dicaffeoylquinic acids chromatography.

Principal component analysis (PCA) was conducted using SPSS 25.0 statistical software to identify key variables contributing to variance in the dataset. Summit areas of the 11 common peaks in the HPLC fingerprints were entered into the rows of the dataset as 11 variables, while the sample numbers were inputs for the columns of the dataset. The results of the analyses showed that the contribution of the cumulative variance of the five principal components reached 90.906 (Table S4), indicating that these five components effectively represented the information from the original eleven variables. Table S4 displays the eigenvalues and contribution rates for the principal components PC1 to PC5. Principal component analysis (PCA) employs orthogonal transformation to convert a set of potentially correlated variables into a set of linearly uncorrelated variables [73]. The first principal component is defined to capture the maximum variance, with each subsequent component capturing the maximum variance possible, while remaining orthogonal to the preceding components [74]. PC1, PC2, PC3, PC4, and PC5 account for 34.009%, 22.028%, 15.205%, 11.856%, and 7.809% of the total variance in the input variables, respectively. These five principal components collectively explain 90.906% of the total variance. To further investigate the contribution and impact of each variable on the principal components, the initial factor loading matrix was calculated. As shown in Table S3, peak 3 has a significant contribution to PC1, peak 5 to PC2, peak 8 to both PC3 and PC4, and peak 10 to PC5. The Mantel test analysis of the common peaks revealed significant correlations, with peak 3 correlating with peaks 6 and 11, and peak 8 correlating with peaks 11, 10, and 2 (Figure 7). The standard verification identified peaks 3 and 8 as chlorogenic acid and 3,5-dicaffeoylquinic acid, respectively (Figure 6B). Consequently, peaks 3 and 8 were used for a further analysis of the chemical composition content, and the representative HPLC chromatogram is shown in Figure 8.

### 2.10. Impact of Light Quality on the Content of Chlorogenic and 3,5-Dicaffeoylquinic Acids

**Figure 9:**
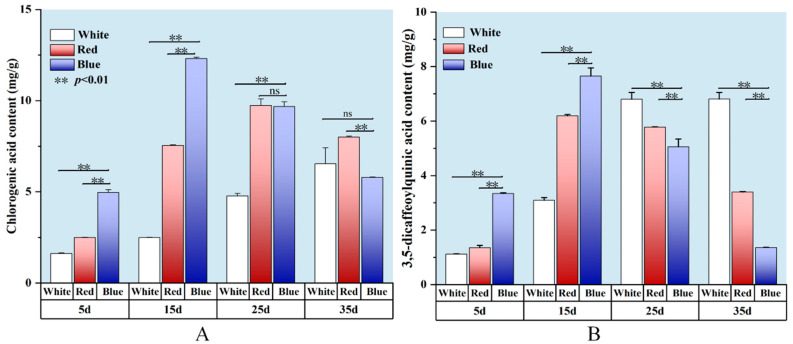
Effects of light qualities on chlorogenic acid and 3,5-di-O-caffeoylquinic acid content in *L. japonica* callus at 5, 15, 25, and 35 days. (**A**) chlorogenic acid content. (**B**) 3,5-di-O-caffeoylquinic acid content. ** indicates extremely significant differences (*p* < 0.01) compared with blue light. ‘ns’ denotes no significant difference (*p* > 0.05).

One of the major medicinal components of *L. japonica* is chlorogenic acid, which is widely used to evaluate the quality of *L. japonica* [75]. The total amount of phenolic acids, including chlorogenic acid, 3,5-di-O-caffeoylquinic acid, and 4,5-di-O-caffeoylquinic acid, serve as a quality control indicator for *L. japonica*. Among them, the contents of chlorogenic acid and 3,5-dicaffeoylquinic acid were affected by different light qualities, as shown in Figure 9.

As depicted in Figure 9A, red and blue light significantly increased the content of phenolic compounds in the *L. japonica* callus than that in white light. Under different light treatments, the chlorogenic acid content of *L. japonica* callus reached the maximum value (12.32 mg g^−1^) at 15 days after induction with blue light, which was 4.9 and 1.6 times higher than that of white light and red light treatments, respectively. The chlorogenic acid content in the blue light treatment increased and then decreased with the incubation time, while the opposite trend was observed in the white light treatment. By the 25th day, the chlorogenic acid content of the red light reached its peak (9.74 mg g^−1^), which was not significantly different from the blue light (*p* > 0.05), but significantly higher than that of white light (*p* < 0.01), being twice as much as in the white light treatment. By day 35, blue light suppressed chlorogenic acid synthesis, while at this time, the chlorogenic acid content under white light reached 6.54 mg g^−1^, which was 12.95% higher than that under blue light.

The light quality and cultivation duration significantly influenced the accumulation of 3,5-di-O-caffeoylquinic acid in *L. japonica* callus (Figure 9B). During the cultivation process, both red and blue light initially promoted and then inhibited 3,5-di-O-caffeoylquinic acid accumulation. On the 5th day of cultivation, blue light and red light increased the accumulation of 3,5-di-O-caffeoylquinic acid by 1.98-fold and 0.21-fold, respectively, compared with white light. By the 15th day, the accumulation of 3,5-di-O-caffeoylquinic acid induced by blue light reached its peak (7.65 mg g^−1^), which was 2.48 times and 1.24 times higher than that under white and red light treatments, respectively. By the 35th day, its content under white light reached 6.81 mg g^−1^, which was five times and two times higher than that under blue and red light treatments, respectively.

In conclusion, blue light is the most effective light quality, significantly promoting the accumulation of chlorogenic acid and 3,5-di-O-caffeoylquinic acid in the *L. japonica* callus, where the optimum incubation time was 15 days. This finding is consistent with the study by Senecović et al., who reported that blue light significantly enhances the accumulation of chlorogenic acid derivatives in the tissue culture of *Bupleurum erectum* [76]. Previous studies have shown that blue light can regulate the expression of genes related to secondary metabolites [77]. For example, in strawberries, blue light significantly upregulated the expression of *FvHCT*, a key gene in the chlorogenic acid biosynthesis pathway, thereby improving chlorogenic acid accumulation [78]. Similarly, in lettuce seedlings, blue light not only enhanced the expression and enzymatic activity of phenylalanine ammonia-lyase (PAL) but also increased the expression levels of cinnamate-4-hydroxylase (C4H) and *p*-coumarate-3-hydroxylase (C3H) [79], thereby promoting chlorogenic acid biosynthesis through the phenylpropanoid pathway.

## 3. Material and Methods

### 3.1. Experiment Materials

Different-sized flower buds of *L. japonica* were collected from the Tongwei Qingliangyuan Company base and authenticated by Professor Yuan Chen (Gansu Agricultural University, Lanzhou, China). The harvested buds were stored in self-sealing bags at 4 °C for 24 h. They were thoroughly washed in running tap water for 30 min, followed by immersion in a 75% (*v*/*v*) ethanol solution for 30 s in an ultra-clean bench. After that, it was rinsed 3–5 times with sterile water, then immersed in 0.1% (*w*/*v*) mercuric chloride solution for 8–10 min, and finally rinsed again 3–5 times with sterile water.

### 3.2. Association Between Flower Bud/Floret Size and Microspore Developmental Stage

To establish the correlation between bud size and microspore development stages, the anthers were dissected from buds of different sizes and then gently squeezed with tweezers to release the contents onto a glass slide. One drop of either acetocarmine solution or 4′,6-diamidino-2-phenylindole (DAPI) staining solution was added, and it was covered with a cover slip. The slide was placed in a dark environment for 30 min. Subsequently, the microspore development period was observed under an optical microscope. (RVL-100-G, ECHO, San Diego, CA, USA).

### 3.3. Callus Induction

This experiment involved the inoculation of sterilized Lonicera japonica anthers into culture bottles containing 50 mL of solid medium with varying concentrations of plant growth regulators (PGRs). Each treatment was repeated 10 times. The basal medium consisted of Murashige & Skoog (MS) medium solidified with 0.5% (*w*/*v*) agar and supplemented with 3% (*w*/*v*) sucrose. To induce callus formation, different concentrations of naphthalene acetic acid (NAA), 2,4-dichlorophenoxyacetic acid (2,4-D), kinetin (KT), and 6-benzyladenine (6-BA) were added. Based on preliminary single-factor experiments, an L_9_ (3^4^) orthogonal design was applied to optimize PGR concentrations. The specific composition of the orthogonal matrix is detailed in Table 2. The culture medium was adjusted to pH 5.8 and sterilized by autoclaving at 121 °C, 1.01 × 10⁵ Pa for 25 min. All cultures were first incubated for 30 days in the dark in an incubator at 20 ± 2 °C and 75% relative humidity, followed by a 45-day photoperiodic culture under a 12-h dark/12-h white fluorescent light (60 μmol m^−2^ s^−1^).

### 3.4. Callus Proliferation

After 75 days of induction, 0.5 g of yellow, fragile callus tissue was transferred onto fresh 50 mL MS medium containing agar, sucrose, and PGRs. The concentrations of PGRs were optimized using an L_9_ (3^4^) orthogonal design, with specific factor levels detailed in Table S1. The medium was adjusted to pH 5.8 and sterilized as described in Section 3.3. All cultures were placed on the racks of the incubator and incubated under photoperiodic conditions at a temperature of 20 ± 2 °C, relative humidity of 75%, and 12-h dark/12-h white fluorescent light (60 μmol m^−2^ s^−1^). The proliferation of healing tissues was observed after 45 days of incubation.

### 3.5. Histomorphological Observation of Callus

#### 3.5.1. Scanning Electron Microscopy (SEM)

Referring to methods of Popielarska-Konieczna et al. [80] and Liu et al. [81], the callus samples in different morphologies were fixed and observed. The samples were fixed in 3% (*w*/*v*) glutaraldehyde solution (0.1 M phosphate buffer, pH 7.2) for 2 h at room temperature. Subsequently, a gradient dehydration process was performed using ethanol solutions with increasing concentrations. The samples were then immersed in tert-butanol at 4 °C, freeze-dried, and mounted on aluminum stubs using conductive adhesive. Prior to scanning electron microscopy (SEM) observation with a Hitachi S-3400N (Japan), the samples were coated with gold, and the accelerating voltage was set to 15 kV.

#### 3.5.2. Paraffin Sections

The histological structure of the callus was observed as previously described [82,83]. The specific steps were as follows: first, the callus was fixed in FAA fixative. Subsequently, the samples were treated with gradient dehydration using different concentrations of ethanol and immersed in a mixture of ethanol and xylene for 2 h. Next, the samples were immersed in a mixture of xylene and paraffin with different ratios, and finally embedded in paraffin blocks. The embedded samples were cut into 7 μm thick tissue sections and stained with safranin O fast green FCF. Finally, the histological structure of the healing tissues was observed using a microscope (Model RVL-100-G, ECHO, San Diego, CA, USA).

### 3.6. Induction of Callus Proliferation Under Different Light Conditions

A total of 1 g of callus was applied to the subculture medium and incubated under three different light quality treatments, namely white light (400–700 nm, 40–50 μmol m^−2^ s^−1^, 100%), blue light (380–560 nm, 25–27 μmol m^−2^ s^−1^, 100%), and red light (610–715 nm, 35–37 μmol m^−2^ s^−1^, 100%). Among them, 100% white light served as the positive control. The incubation temperature was 25 ± 1 °C, and the relative humidity was 60–75%. The photoperiod for each light quality treatment is 12 h of light and 12 h of darkness. There were three biological replicates for each treatment (light quality × cultivation time (5, 15, 25 or 35 days). The callus cultures were taken out from three culture vessels, representing each replicate, and frozen in liquid nitrogen. Then, a mortar and pestle were used to grind the callus into powder. Each powder was dried in a vacuum freeze dryer (temperature of −50 °C and vacuum of 25 Pa) and stored at −80 °C until analysis.

### 3.7. Phytochemical Analysis

For each callus culture, 0.15 g of the freeze-dried powder was placed into a 50 mL centrifuge tube, followed by the addition of 30 mL of 75% (*v*/*v*) methanol. Subsequently, the sample was subjected to ultrasonic disruption for 10 min and then oscillated on an oscillator at 150 rpm and 25 °C for 30 min. After that, it was centrifuged at 5000 rpm for 10 min. The supernatant was collected, and the aforementioned steps were repeated for two additional extractions. Finally, the supernatant was transferred to a 10 mL centrifuge tube and stored in a refrigerator at 4 °C.

#### 3.7.1. Determination of Total Flavonoid, Total Phenolic Content and Soluble Sugar

##### Total Flavonoid Content

The total flavonoid content was determined by the sodium nitrite–aluminum nitrate–sodium hydroxide colorimetric method [84,85]. The specific procedure was as follows: 600 μL of the extract was mixed with a reaction solution (distilled water, 5% NaNO_2_,10% AlCl_3_). The absorbance was measured at 510 nm using a UV-Vis spectrophotometer (Thermo Fisher Scientific, Genesys 10S UV-Vis, Waltham, MA, USA). Catechin (0.2–1.0 mg mL^−1^) was used as the standard to construct the calibration curve. The final results were expressed as mg catechin equivalent (mg CE) g^−1^ dry weight (g^−1^ DW).

##### Total Phenolic Content

The total phenol content was determined using the Folin–Ciocalteu colorimetric method [86,87]. The procedure was as follows: 600 μL of the extract was added to the reaction mixture (10% Folin–Ciocalteu reagent, 7.5% Na_2_CO_3_), followed by incubation in a water bath at 37 °C for 1 h in the dark. Subsequently, the absorbance was measured at 760 nm using a UV-Vis spectrophotometer. A calibration curve was drawn using gallic acid (0.02–0.1 mg mL^−1^) as the standard, and the total phenol content was expressed as milligrams gallic acid equivalent (mg GAE) g^−1^ dry weight (g^−1^ DW).

##### Soluble Sugar Content

The soluble sugar content was measured by the sulfuric acid–phenol method [88]. The specific procedure was as follows: 500 μL of extract was added to the reaction mixture and incubated at room temperature for 30 min. The absorbance was then measured at 485 nm using a UV-Vis spectrophotometer. A calibration curve was constructed using sucrose (0.05–0.6 mg mL^−1^) as the standard. The soluble sugar content was expressed as mg sucrose equivalent (mg SSE) g^−1^ dry weight (g^−1^ DW).

#### 3.7.2. Determination of Antioxidant Capacity

Two widely used methods, namely the DPPH method and the FRAP method [89], have been employed for the assessment of in vitro antioxidant capacity. In the DPPH assay, 500 μL of the extract was added to the DPPH methanol solution according to the established protocol [90], and the absorbance was measured at 515 nm using a UV-visible spectrophotometer after shaking for 30 min at room temperature protected from light. For the FRAP assay, following a standard method [91,92], 500 μL of extract was added to the FRAP reaction mixture and incubated in a water bath at 37 °C for 4 min. The absorbance was then measured at 593 nm using a UV-Vis spectrophotometer. A calibration curve was constructed using a 500 μM methanolic solution of 90% ascorbic acid as the reference standard.

### 3.8. HPLC Fingerprint Establishment

#### 3.8.1. Chromatographic Conditions

HPLC fingerprint analysis was conducted using a Waters H-Class HPLC system (Milford, MA, USA). Sample separation was performed on a Waters Symmetry C18 column (250 × 4.6 mm, 5 μm) with a 50 min gradient elution using a mobile phase of acetonitrile (A) and 0.1% phosphoric acid solution (B) at a flow rate of 1.0 mL min^−1^. The gradient program was as follows: initial A at 9%; 10–20 min, A increased to 15–17.5%; 20–30 min, A adjusted to 17.5–17.8%; 30–40 min, A raised to 17.8–22%; and 40–50 min, A returned to 9%. The detection wavelength was set at 245 nm for fingerprint analysis. The column temperature was maintained at 38 °C, and the injection volume was 10 μL.

#### 3.8.2. Fingerprint Establishment

To develop the UPLC fingerprint, chromatographic conditions were optimized, and the method was validated in a previous study [93]. During validation, precision was assessed by performing six consecutive injections of the same sample solution, while repeatability was evaluated by analyzing six independently prepared sample solutions. Stability was tested by measuring sample solutions at 0, 2, 4, 8, 16, and 24 h. Chromatographic data were processed using the “Traditional Chinese Medicine Chromatographic Fingerprint Similarity Evaluation System (2012 edition)”, a specialized software recommended by the China National Food and Drug Administration [94]. The software determined the chromatogram similarity by calculating the correlation coefficient and the cosine of the vector angle. A similarity value above 0.9 indicated a strong correlation, classifying the samples as highly similar.

### 3.9. Statistical Analysis

The data are expressed as mean ± standard error (SE, *n* = 3) and were computed using Microsoft Excel 2019. Statistical analyses, including an analysis of variance (ANOVA), were carried out using SPSS version 20. The graphical representation of the results was performed with OriginPro 2021 and R Studio 4.0.4.

## 4. Conclusions

This study successfully induced embryogenic callus tissues from the anthers of *L. japonica* for the first time and investigated the effects of light induction on callus morphology, metabolite accumulation, and antioxidant activity. The results showed that the MS medium supplemented with 1.0 mg·L^−1^ 6-BA, 1.5 mg·L^−1^ NAA, 1.5 mg·L^−1^ 2,4-D, and 0.2 mg·L^−1^ KT efficiently induced an 89% embryogenic callus formation. In subsequent subcultures, the use of 2.0 mg·L^−1^ 6-BA, 0.5 mg·L^−1^ NAA, and 0.2 mg·L^−1^ KT successfully resulted in a uniform callus. Morphological observations revealed that the surface of the embryogenic callus cells exhibited closely arranged spherical protrusions, with distinct nuclei and abundant starch grains, indicating strong differentiation potential. Light induction experiments showed that the blue light treatment significantly promoted metabolite accumulation in the callus, particularly at 15 days, when metabolites (total phenols, flavonoids, soluble sugars, chlorogenic acid, and 3,5-dicaffeoylquinic acid) and antioxidant activity (DPPH, FRAP) peaked. Overall, the results of this study indicate that an appropriate plant hormone combination effectively promotes the formation of *L. japonica* anther-derived callus, and light treatment significantly enhances the accumulation of metabolites and antioxidant activity. This study provides new insights for the germplasm innovation of *L. japonica* and demonstrates the potential of light induction and abiotic inducers to enhance the yield of secondary metabolites in medicinal plants.

## Figures and Tables

**Table 1:** Effect of microspore developmental on callus induction in *L. japonica*.

Stage	Induction Rate (%)	Browning Rate (%)	Contamination Rate (%)
Tetrad stage	13 ± 0.40 ^c^	72 ± 0.29 ^a^	23 ± 0.84 ^a^
Early uninucleate stage	38 ± 0.70 ^b^	52 ± 0.89 ^b^	10 ± 0.37 ^ab^
Late-uninucleate stage	78 ± 0.29 ^a^	15 ± 0.27 ^c^	7 ± 0.30 ^b^
Binucleate stage	12 ± 0.25 ^c^	85 ± 0.31 ^a^	3 ± 0.15 ^b^

Values within each column are marked with different letters a indicate significant differences, as determined by Duncan’s multiple range test (*p* < 0.05).

**Table 2:** Effect of NAA, 2,4-D, KT, and 6-BA variables on anther-induced callus.

Test No.	A(6-BA) mg·L^−1^	B(NAA) mg·L^−1^	C(2,4-D) mg·L^−1^	D(KT) mg·L^−1^	Induction Rate (%)	Callus Characteristics
I	1(0.5)	1(0.5)	1(0.5)	1(0.2)	17 ± 0.30 ^e^	Yellowish-white, watery
II	1(0.5)	2(1.0)	3(1.5)	2(0.5)	49 ± 0.38 ^c^	Light brown, loose
III	1(0.5)	3(1.5)	2(1.0)	3(0.8)	69 ± 0.38 ^b^	green, slightly brown, less friable
IV	2(1.0)	1(0.5)	3(1.5)	3(0.8)	31 ± 0.31 ^d^	Yellow-white, loose
V	2(1.0)	2(1.0)	2(1.0)	1(0.2)	64 ± 0.34 ^b^	White-green, slightly brown, compact
VI	2(1.0)	3(1.5)	1(0.5)	2(0.5)	81 ± 0.23 ^a^	Yellow, friable
VII	3(1.5)	1(0.5)	2(1.0)	2(0.5)	9 ± 0.23 ^e^	White-brown, slightly watery
VIII	3(1.5)	2(1.0)	1(0.5)	3(0.8)	18 ± 0.25 ^e^	Yellowish-white, slightly watery
IX	3(1.5)	3(1.5)	3(1.5)	1(0.2)	70 ± 0.33 ^b^	White-green, compact

Values within each column are marked with different letters a indicate significant differences, as determined by Duncan’s multiple range test (*p* < 0.05).

**Table 3:** ANOVA and range analyses were conducted on callus induced from anthers using the L_9_ (3^4^) orthogonal experiment.

Analytical Method	A(6-BA) mg·L^−1^	B(NAA) mg·L^−1^	C(2,4-D) mg·L^−1^	D(KT) mg·L^−1^
ANOVA analysis				
Df	2	2	2	2
MS	0.52	2.22	0.105	0.093
*p* value	0.0001 **	0.0001 **	0.0001 **	0.0001 **
Range analysis				
k1	0.45	0.19	0.39	0.50
k2	0.59	0.44	0.47	0.46
k3	0.32	0.73	0.50	0.39
Rk	0.27	0.54	0.11	0.11
Best level	A_2_	B_3_	C_3_	D_1_

ki represents the average response of each factor at different levels where i denotes a level and k denotes a factor. Rk = maxki − minki for each factor. NAA, naphthalene acetic acid; 2,4-D, 2,4-dichlorophenoxy acetic acid; 6-BA, 6–benzyladenine; KT, kinetin. A value of 1 corresponds to a low level in the orthogonal design. A value of 2 corresponds to a middle level in the orthogonal design. A value of 3 corresponds to a high level in the orthogonal design; ** indicates extremely significant differences (*p* < 0.01).

**Table 4:** The similarity values of *L. japonica* in different light qualities.

NO.	S1	S2	S3	S4	S5	S6	S7	S8	S9	S10	S11	S12	R
S1	1												
S2	0.976	1											
S3	0.984	0.982	1										
S4	0.983	0.987	0.995	1									
S5	0.935	0.907	0.896	0.919	1								
S6	0.972	0.929	0.962	0.955	0.939	1							
S7	0.843	0.805	0.808	0.833	0.928	0.856	1						
S8	0.956	0.985	0.975	0.977	0.856	0.897	0.755	1					
S9	0.956	0.948	0.93	0.946	0.922	0.896	0.776	0.936	1				
S10	0.934	0.969	0.955	0.953	0.803	0.859	0.722	0.992	0.902	1			
S11	0.939	0.972	0.958	0.959	0.822	0.868	0.723	0.996	0.92	0.997	1		
S12	0.843	0.77	0.822	0.805	0.861	0.913	0.847	0.704	0.721	0.658	0.653	1	
R	0.991	0.975	0.983	0.987	0.959	0.979	0.887	0.95	0.943	0.921	0.927	0.872	1

## Data Availability

Date will be made available on request.

## Supplementary Materials

The following supporting information can be downloaded at: https://www.mdpi.com/article/10.3390/ijms26052351/s1.

## References

[1] Zhu W., Zheng W., Hu X., Xu X., Zhang L., Tian J. (2017). Variations of Metabolites and Proteome in *Lonicera Japonica Thunb*. Buds and Flowers under UV Radiation. Biochim. Biophys. Acta BBA-Proteins Proteom..

[2] Li Y., Li W., Fu C., Song Y., Fu Q. (2020). *Lonicerae japonicae* Flos and *Lonicerae* Flos: A Systematic Review of Ethnopharmacology, Phytochemistry and Pharmacology. Phytochem. Rev..

[3] Kang O.H., Choi Y.A., Park H.J., Lee J.Y., Kim D.K., Choi S.C., Kim T.H., Nah Y.H., Yun K.J., Choi S.J. (2004). Inhibition of Trypsin-Induced Mast Cell Activation by Water Fraction of *Lonicera japonica*. Arch. Pharm. Res..

[4] Park H.S., Park K.I., Lee D.H., Kang S.R., Nagappan A., Kim J.A., Kim E.H., Lee W.S., Shin S.C., Hah Y.S. (2012). Polyphenolic Extract Isolated from Korean *Lonicera japonica* Thunb. Induce G2/M Cell Cycle Arrest and Apoptosis in HepG2 Cells: Involvements of PI3K/Akt and MAPKs. Food Chem. Toxicol..

[5] Ryu K.H., Rhee H.I., Kim J.H., Yoo H., Lee B.Y., Um K.A., Kim K., Noh J.Y., Lim K.M., Chung J.H. (2010). Anti-Inflammatory and Analgesic Activities of SKLJI, a Highly Purified and Injectable Herbal Extract of *Lonicera japonica*. Biosci. Biotechnol. Biochem..

[6] Zhang F., Shi P., Liu H., Zhang Y., Yu X., Li J., Pu G.A. (2019). Simple, Rapid, and Practical Method for Distinguishing *Lonicerae Japonicae* Flos from *Lonicerae* Flos. Molecules.

[7] Zhang M., Xiao Q., Li Y., Tian Y., Zheng J., Zhang J. (2024). Exploration of Exogenous Chlorogenic Acid as a Potential Plant Stimulant: Enhancing Physiochemical Properties in *Lonicera japonica*. Physiol. Mol. Biol. Plants.

[8] Sun C., Teng Y., Li G., Yoshioka S., Yokota J., Miyamura M., Fang H., Zhang Y. (2010). Metabonomics Study of the Protective Effects of *Lonicera Japonica* Extract on Acute Liver Injury in Dimethylnitrosamine Treated Rats. J. Pharm. Biomed. Anal..

[9] Wang T., Yang B., Guan Q., Chen X., Zhong Z., Huang W., Zhu W., Tian J. (2019). Transcriptional Regulation of *Lonicera japonica* Thunb. during Flower Development as Revealed by Comprehensive Analysis of Transcription Factors. BMC Plant Biol..

[10] Dias M.C., Pinto D.C.G.A., Silva A.M.S. (2021). Plant Flavonoids: Chemical Characteristics and Biological Activity. Molecules.

[11] Chen L., Cao H., Huang Q., Xiao J., Teng H. (2022). Absorption, Metabolism and Bioavailability of Flavonoids: A Review. Crit. Rev. Food Sci. Nutr..

[12] Fang Z., Li J., Yang R., Fang L., Zhang Y. (2020). A Review: The Triterpenoid Saponins and Biological Activities of *Lonicera* Linn. Molecules.

[13] Lai K.H., Chen Y.L., Lin M.F., El-Shazly M., Chang Y.C., Chen P.J., Su C.H., Chiu Y.C., Illias A.M., Chen C.C. (2022). *Lonicerae japonicae* Flos Attenuates Neutrophilic Inflammation by Inhibiting Oxidative Stress. Antioxidants.

[14] Hu M., Hu Z., Du L., Du J., Luo Q., Xiong J. (2019). Establishment of Cell Suspension Culture of *Lonicera japonica* Thunb and Analysis Its Major Secondary Metabolites. Ind. Crops Prod..

[15] Pan Y., Li L., Xiao S., Chen Z., Sarsaiya S., Zhang S., Guang S., Liu H. (2020). Callus growth kinetics and accumulation of secondary metabolites of *Bletilla striata* Rchb.f. using a callus suspension culture. PLoS ONE..

[16] Buranasudja V., Rani D., Malla A., Kobtrakul K., Vimolmangkang S. (2021). Insights into Antioxidant Activities and Anti-Skin-Aging Potential of Callus Extract from *Centella asiatica* (L.). Sci. Rep..

[17] Yang L., Wen K.S., Ruan X., Zhao Y.X., Wei F., Wang Q. (2018). Response of Plant Secondary Metabolites to Environmental Factors. Molecules.

[18] Betekhtin A., Rojek M., Nowak K., Pinski A., Milewska-Hendel A., Kurczynska E., Doonan J.H., Hasterok R. (2018). Cell Wall Epitopes and Endoploidy as Reporters of Embryogenic Potential in *Brachypodium distachyo* Callus Culture. Int. J. Mol. Sci..

[19] Lv S., Ding F., Zhang S., Nosov A.M., Kitashov A.V., Yang L. (2024). Induction and Suspension Culture of *Panax japonicus* Callus Tissue for the Production of Secondary Metabolic Active Substances. Plants.

[20] Bondarev N., Reshetnyak O., Bondareva T., Il’in M., Nosov N. (2019). Impact of cultivation factors in vitro on the growth and the biosynthesis of steviol glycosides in Stevia rebaudiana cell cultures. Physiol. Mol. Biol. Plants.

[21] Espinosa-Leal C.A., Puente-Garza C.A., García-Lara S. (2018). In vitro plant tissue culture: Means for production of biological active compounds. Planta.

[22] Huang P., Xia L., Zhou L., Liu W., Wang P., Qing Z., Zeng J. (2021). Influence of Different Elicitors on BIA Production in *Macleaya cordata*. Sci. Rep..

[23] Akula R., Ravishankar G.A. (2011). Influence of Abiotic Stress Signals on Secondary Metabolites in Plants. Plant Signal. Behav..

[24] Tariq U., Ali M., Abbasi B.H. (2014). Morphogenic and Biochemical Variations under Different Spectral Lights in Callus Cultures of *Artemisia absinthium* L. J. Photochem. Photobiol. B.

[25] Sytar O., Zivcak M., Neugart S., Toutounchi P.M., Brestic M. (2019). Precultivation of young seedlings under different color shades modifies the accumulation of phenolic compounds in Cichorium leaves in later growth phases. Environ. Exp. Bot..

[26] Dantas L.A., Rosa M., Resende E.C., Silva F.G., Pereira P.S., Souza A.C.L., e Silva F.H.L., Neto A.R. (2020). Spectral quality as an elicitor of bioactive compound production in *Solanum aculeatissimum* JACQ cell suspension. J. Photochem. Photobiol. B.

[27] Sobhani Najafabadi A., Khanahmadi M., Ebrahimi M., Moradi K., Behroozi P., Noormohammadi N. (2019). Effect of different quality of light on growth and production of secondary metabolites in adventitious root cultivation of *Hypericum perforatum*. Plant Signal. Behav..

[28] Ahmad N., Rab A., Ahmad N. (2016). Light-Induced Biochemical Variations in Secondary Metabolite Production and Antioxidant Activity in Callus Cultures of *Stevia Rebaudiana* (Bert). J. Photochem. Photobiol. B.

[29] Fazal H., Abbasi B.H., Ahmad N., Ali S.S., Akbar F., Kanwal F. (2016). Correlation of Different Spectral Lights with Biomass Accumulation and Production of Antioxidant Secondary Metabolites in Callus Cultures of Medicinally Important *Prunella vulgaris* L. J. Photochem. Photobiol. B.

[30] Lai C.C., Pan H., Zhang J., Wang Q., Que Q.X., Pan R., Lai Z.X., Lai G.T. (2022). Light Quality Modulates Growth, Triggers Differential Accumulation of Phenolic Compounds, and Changes the Total Antioxidant Capacity in the Red Callus of *Vitis davidii*. J. Agric. Food Chem..

[31] Hayta S., Gurel A., Akgun I.H., Altan F., Ganzera M., Tanyolac B., Bedir E. (2011). Induction of *Gentiana cruciata* Hairy Roots and Their Secondary Metabolites. Biologia.

[32] Singh J., Sabir F., Sangwan R.S., Narnoliya L.K., Saxena S., Sangwan N.S. (2015). Enhanced Secondary Metabolite Production and Pathway Gene Expression by Leaf Explants-Induced Direct Root Morphotypes Are Regulated by Combination of Growth Regulators and Culture Conditions in *Centella asiatica* (L.) Urban. Plant Growth Regul..

[33] Azeez H., Ibrahim K., Pop R., Pamfil D., Hârţa M., Bobiș O. (2017). Changes Induced by Gamma Ray Irradiation on Biomass Production and Secondary Metabolites Accumulation in *Hypericum Triquetrifolium* Turra Callus Cultures. Ind. Crops Prod..

[34] Karakas F.P. (2020). Efficient Plant Regeneration and Callus Induction from Nodal and Hypocotyl Explants of Goji Berry (*Lycium barbarum* L.) and Comparison of Phenolic Profiles in Calli Formed under Different Combinations of Plant Growth Regulators. Plant Physiol. Biochem..

[35] Maharani A., Fanata W.I.D., Laeli F.N., Kim K.M., Handoyo T. (2020). Callus Induction and Regeneration from Anther Cultures of Indonesian Indica Black Rice Cultivar. J. Crop Sci. Biotechnol..

[36] Germanà M.A. (2011). Anther Culture for Haploid and Doubled Haploid Production. Plant Cell Tissue Organ Cult..

[37] Bhatia R., Dey S.S., Sood S., Sharma K., Sharma V.K., Parkash C., Kumar R. (2016). Optimizing Protocol for Efficient Microspore Embryogenesis and Doubled Haploid Development in Different Maturity Groups of Cauliflower (*B. oleracea* Var. *Botrytis* L.) in India. Euphytica.

[38] Kumar K.R., Singh K.P., Bhatia R., Raju D.V.S., Panwar S. (2019). Optimising Protocol for Successful Development of *Haploids* in Marigold (*Tagetes* spp.) through in Vitro Androgenesis. Plant Cell Tissue Organ Cult..

[39] Alan A.R., Celebi-Toprak F., Lachin A., Yildiz D., Gozen V., Besirli G., Segui-Simarro J.M. (2021). Doubled Haploid Broccoli (*Brassica olearacea* Var. Italica) Plants from Anther Culture. Doubled Haploid Technology: Volume 2: Hot Topics, Apiaceae, Brassicaceae, Solanaceae.

[40] Baliyan N., Srivastava A., Rao M., Mishra A.K., Bharti H., Khar A., Mangal M. (2024). Correlation of Stages of Microsporogenesis with Bud and Anther Morphology in Pepper Genotypes through DAPI Staining with Different Levels of Mordant in *Cytological fixative*. Protoplasma.

[41] Yang K., Zhou X., Wang Y., Feng H., Ren X., Liu H., Liu W. (2017). Carbohydrate Metabolism and Gene Regulation during Anther Development in an Androdioecious Tree, *Tapiscia sinensis*. Ann. Bot..

[42] Yuliani F., Dewi W.S., Yunus A., Siswanto U. (2018). The study of artemisinin content in callus *Artemisia annua* L. cultures elicited with endophytic *Fungi aspergillus* sp. Molecules.

[43] Zayova E., Nedev T., Petrova D., Zhiponova M., Kapchina V., Chaneva G. (2020). Tissue Culture Applications of *Artemisia annua* L. Callus for Indirect Organogenesis and Production Phytochemical. Plant Tissue Cult. Biotechnol..

[44] Iiyama C.M., Sartoratto A., Cardoso J.C. (2024). Tissue-Specific and Novel Secondary Metabolites Identified by SPME/GC-MS in *Artemisia annua* Calli Cultured under Different Wavelength. Ind. Crops Prod..

[45] Wu S., Zu Y., Wu M. (2003). High Yield Production of Salidroside in the Suspension Culture of *Rhodiola sachalinensis*. J. Biotechnol..

[46] Rout P., Naik N., Ngangkham U., Verma R.L., Katara J.L., Singh O.N., Samantaray S. (2016). Doubled Haploids Generated through Anther Culture from an Elite Long Duration Rice Hybrid, CRHR32: Method Optimization and Molecular Characterization. Plant Biotechnol..

[47] Jamil S.Z.M.R., Rohani E.R., Baharum S.N., Noor N.M. (2018). Metabolite Profiles of Callus and Cell Suspension Cultures of *Mangosteen*. 3 Biotech..

[48] Efferth T. (2019). Biotechnology Applications of Plant Callus Cultures. Engineering.

[49] Ren X., Liu Y., Jeong B.R. (2020). Callus Induction and Browning Suppression in Tree Peony *Paeonia ostii* ‘Fengdan’. Hortic. Environ. Biotechnol..

[50] Dai L., Han S., Zhang Y., Hao D. (2022). Genetic Architecture of Embryogenic Callus Induction in Maize from the Perspective of Population Genomics. Plant Cell Tissue Organ Cult..

[51] Binte Mostafiz S., Wagiran A. (2018). Efficient Callus Induction and Regeneration in Selected Indica Rice. Agronomy.

[52] Corral-Martínez P., Siemons C., Horstman A., Angenent G.C., de Ruijter N., Boutilier K. (2020). Live Imaging of Embryogenic Structures in *Brassica Napus* Microspore Embryo Cultures Highlights the Developmental Plasticity of Induced Totipotent Cells. Plant Reprod..

[53] Zhang M., Wang A., Qin M., Qin X., Yang S., Su S., Sun Y., Zhang L. (2021). Direct and Indirect Somatic Embryogenesis Induction in *Camellia oleifera* Abel. Front. Plant Sci..

[54] Yu Y., Yang B., Ma D., Guo S., Liao F., Li Z. (2024). Study on the Induction of Somatic Embryogenesis and Morphological Structural Changes during the Development of *Handeliodendron bodinieri* L. Plant Cell Tissue Organ Cult..

[55] Kong E.Y.Y., Biddle J., Kalaipandian S., Adkins S.W. (2023). Coconut Callus Initiation for Cell Suspension Culture. Plants.

[56] Huang T., Liu D., Cui X., Li M., Jin L., Paré P.W., Li M., Wei J. (2023). In Vitro Bioactive Metabolite Production and Plant Regeneration of Medicinal Plant *Angelica sinensis*. Ind. Crops Prod..

[57] Fang H., Dong Y., Zhou R., Wang Q., Duan Q., Wang C., Bao Y., Xu S., Lang X., Gai S. (2022). Optimization of the Induction, Germination, and Plant Regeneration System for Somatic Embryos in Apomictic Walnut (*Juglans regia* L.). Plant Cell Tissue Organ Cult..

[58] Zhao X., Lian Y., Jin Z., Zhang X., Yan Y., Fan S. (2022). Shoot Organogenesis and Somatic Embryogenesis in Leaf Tissue of *Pulsatilla tongkangensis* Y.N. Lee & T.C. Lee. Plant Biotechnol. Rep..

[59] Mandal S.M., Dias R.O., Franco O.L. (2017). Phenolic Compounds in Antimicrobial Therapy. J. Med. Food.

[60] Foss K., Przybyłowicz K.E., Sawicki T. (2022). Antioxidant Activity and Profile of Phenolic Compounds in Selected Herbal Plants. Plant Foods Hum. Nutr..

[61] Yu J., Liu X., Wang W., Zhang L., Wang C., Zhang Q., Wang J., Du M., Sheng L., Hu D. (2024). MdCIbHLH1 Modulates Sugar Metabolism and Accumulation in Apple Fruits by Coordinating Carbohydrate Synthesis and Allocation. Hortic. Plant J..

[62] Julius B.T., Slewinski T.L., Baker R.F., Tzin V., Zhou S., Bihmidine S., Jander G., Braun D.M. (2018). Maize Carbohydrate Partitioning Defective1 Impacts Carbohydrate Distribution, Callose Accumulation, and Phloem Function. J. Exp. Bot..

[63] Kapoor S., Raghuvanshi R., Bhardwaj P., Sood H., Saxena S., Chaurasia O.P. (2018). Influence of Light Quality on Growth, Secondary Metabolites Production and Antioxidant Activity in Callus Culture of *Rhodiola imbricata* Edgew. J. Photochem. Photobiol. B.

[64] Fan X., Zang J., Xu Z., Guo S., Jiao X., Liu X., Gao Y. (2013). Effects of Different Light Quality on Growth, Chlorophyll Concentration and Chlorophyll Biosynthesis Precursors of Non-Heading Chinese Cabbage (*Brassica campestris* L.). Acta Physiol. Plant..

[65] Hassanpour H. (2022). Potential Impact of Red-Blue LED Light on Callus Growth, Cell Viability, and Secondary Metabolism of *Hyoscyamus reticulatus*. In Vitro Cell. Dev. Biol.-Plant..

[66] Zhang X., Bian Z., Li S., Chen X., Lu C. (2019). Comparative Analysis of Phenolic Compound Profiles, Antioxidant Capacities, and Expressions of Phenolic Biosynthesis-Related Genes in Soybean Microgreens Grown under Different Light Spectra. J. Agric. Food Chem..

[67] Kedare S.B., Singh R.P. (2011). Genesis and Development of DPPH Method of Antioxidant Assay. J. Food Sci. Technol..

[68] Nazir M., Ullah M.A., Younas M., Siddiquah A., Shah M., Giglioli-Guivarc’h N., Hano C., Abbasi B.H. (2020). Light-Mediated Biosynthesis of Phenylpropanoid Metabolites and Antioxidant Potential in Callus Cultures of Purple Basil (*Ocimum basilicum* L. Var Purpurascens). Plant Cell Tissue Organ Cult..

[69] Biswal B., Jena B., Giri A.K., Acharya L. (2022). Monochromatic Light Elicited Biomass Accumulation, Antioxidant Activity, and Secondary Metabolite Production in Callus Culture of *Operculina turpethum* (L.). Plant Cell Tissue Organ Cult..

[70] Azad M.O., Kim W.W., Park C.H., Cho D.H. (2018). Effect of Artificial LED Light and Far Infrared Irradiation on Phenolic Compound, Isoflavones and Antioxidant Capacity in Soybean (*Glycine max* L.) Sprout. Foods.

[71] Liu J., Liu H., Dai Z., Ma S. (2018). Quality Analysis of Long Dan Xie Gan Pill by a Combination of Fingerprint and Multicomponent Quantification with Chemometrics Analysis. J. Anal. Methods Chem..

[72] Wang Z., Jia Y., Li P., Tang Z., Guo Y., Wen L., Yu H., Cui F., Hu F. (2023). Study on Environmental Factors Affecting the Quality of *Codonopsis Radix* Based on MaxEnt Model and All-in-One Functional Factor. Sci. Rep..

[73] Liang X., Guo Z.C., Wang L., Li R.C., Lin W.W. (2023). Nearly Optimal Stochastic Approximation for Online Principal Subspace Estimation. Sci. China Math..

[74] Luo G., Chen G., Tian L., Qin K., Qian S.E. (2016). Minimum Noise Fraction versus Principal Component Analysis as a Preprocessing Step for Hyperspectral Imagery Denoising. Can. J. Remote Sens..

[75] Chinese Pharmacopoeia Commission (2020). Pharmacopoeia of the People’s Republic of China.

[76] Senekovič J., Ciringer T., Ambrožič-Dolinšek J., Islamčević Razboršek M. (2024). The Effect of Combined Elicitation with Light and Temperature on the Chlorogenic Acid Content, Total Phenolic Content and Antioxidant Activity of *Berula erecta* in Tissue Culture. Plants.

[77] Nam T.G., Kim D.O., Eom S.H. (2018). Effects of Light Sources on Major Flavonoids and Antioxidant Activity in Common Buckwheat Sprouts. Food Sci. Biotechnol..

[78] Chen X., Cai W., Xia J., Yu H., Wang Q., Pang F., Zhao M. (2020). Metabolomic and Transcriptomic Analyses Reveal That Blue Light Promotes Chlorogenic Acid Synthesis in Strawberry. J. Agric. Food Chem..

[79] Endo M., Fukuda N., Yoshida H., Fujiuchi N., Yano R., Kusano M. (2022). Effects of Light Quality, Photoperiod, CO_2_ Concentration, and Air Temperature on Chlorogenic Acid and Rutin Accumulation in Young Lettuce Plants. Plant Physiol. Biochem..

[80] Popielarska-Konieczna M., Kozieradzka-Kiszkurno M., Świerczyńska J., Góralski G., Ślesak H., Bohdanowicz J. (2008). Ultrastructure and Histochemical Analysis of Extracellular Matrix Surface Network in Kiwifruit Endosperm-Derived Callus Culture. Plant Cell Rep..

[81] Liu X., Zhao Y., Chen X., Dong L., Zheng Y., Wu M., Ding Q., Xu S., Ding C., Liu W. (2021). Establishment of Callus Induction System, Histological Evaluation and Taxifolin Production of Larch. Plant Cell Tissue Organ Cult..

[82] Li M., Lv M., Yang D., Wei J., Xing H., Paré P.W. (2020). Temperature-Regulated Anatomical and Gene-Expression Changes in *Sinopodophyllum hexandrum* Seedlings. Ind. Crops Prod..

[83] Liu X., Luo M., Li M., Wei J. (2022). Depicting Precise Temperature and Duration of Vernalization and Inhibiting Early Bolting and Flowering of *Angelica sinensis* by Freezing Storage. Front. Plant Sci..

[84] Yao W., Lei D., Zhou X., Wang H., Lu J., Lin Y., Zhang Y., Wang Y., He W., Li M. (2023). Effect of Different Culture Conditions on Anthocyanins and Related Genes in Red Pear Callus. Agronomy.

[85] Lay M.M., Karsani S.A., Mohajer S., Abd Malek S.N. (2014). Phytochemical Constituents, Nutritional Values, Phenolics, Flavonols, Flavonoids, Antioxidant and Cytotoxicity Studies on *Phaleria macrocarpa* (Scheff.) Boerl Fruits. BMC Complement. Altern. Med..

[86] Han Z., Zhang J., Cai S., Chen X., Quan X., Zhang G. (2018). Association Mapping for Total Polyphenol Content, Total Flavonoid Content and Antioxidant Activity in Barley. BMC Genom..

[87] Villamarin-Raad D.A., Lozano-Puentes H.S., Chitiva L.C., Costa G.M., Díaz-Gallo S.A., Díaz-Ariza L.A. (2023). Changes in Phenolic Profile and Total Phenol and Total Flavonoid Contents of Guadua *Angustifolia kunth* Plants under Organic and Conventional Fertilization. ACS Omega.

[88] Chow P.S., Landhäusser S.M. (2004). A Method for Routine Measurements of Total Sugar and Starch Content in Woody Plant Tissues. Tree Physiol..

[89] Thaipong K., Boonprakob U., Crosby K., Cisneros-Zevallos L., Byrne D.H. (2006). Comparison of ABTS, DPPH, FRAP, and ORAC Assays for Estimating Antioxidant Activity from Guava Fruit Extracts. J. Food Compos. Anal..

[90] Jha D.K., Panda L., Ramaiah S., Anbarasu A. (2014). Evaluation and Comparison of Radical Scavenging Properties of Solvent Extracts from *Justicia Adhatoda* Leaf Using DPPH Assay. Appl. Biochem. Biotechnol..

[91] Sethi S., Joshi A., Arora B., Bhowmik A., Sharma R.R., Kumar P. (2020). Significance of FRAP, DPPH, and CUPRAC Assays for Antioxidant Activity Determination in Apple Fruit Extracts. Eur. Food Res. Technol..

[92] Chaves N., Santiago A., Alías J.C. (2020). Quantification of the Antioxidant Activity of Plant Extracts: Analysis of Sensitivity and Hierarchization Based on the Method Used. Antioxidants.

[93] Cheng J., Guo F., Wang L., Li Z., Zhou C., Wang H., Liang W., Jiang X., Chen Y., Dong P. (2024). Evaluating the impact of ecological factors on the quality and habitat distribution of *Lonicera japonica* Flos using HPLC and the MaxEnt model. Front. Plant Sci..

[94] Jin Y., Liang T., Fu Q., Xiao Y.S., Feng J.T., Ke Y.X., Liang X.M. (2009). Fingerprint analysis of *Ligusticum chuanxiong* using hydrophilic interaction chromatography and reversed-phase liquid chromatography. J. Chromatogr. A.

