# Supplementary material for: Callus Culture System from Lonicera japonica Thunb Anthers: Light Quality Effects on Callus Quality Evaluation
Source: Int J Mol Sci. 2025 Mar 6;26(5):2351. doi: 10.3390/ijms26052351 (PMC11900127; doi:10.3390/ijms26052351)
Supplement: Supplementary file 1 [file ijms-26-02351-s001.zip › ijms-3440128-supplementary.pdf]

Callus Culture System from *Lonicera japonica Thunb* Anthers: Light Quality Effects on Callus Quality Evaluation

Jiali Cheng [1], Fengxia Guo [1], Chunyan Zhou [2], Wei Liang [1], Yong Chen [3], Hongyan Wang [1], Yuan Chen [1*] and Pengbin Dong [1*]

[1] State Key Laboratory of Aridland Crop Science, College of Agronomy, College of Life Science and Technology, Gansu Agricultural University, Lanzhou 730070, China

[2] College of Economics and Management, Hexi University, Zhangye, 734000, China

[3] Institute of Soil, Fertilizer and Agricultural Water saving, Xinjiang Academy of Agricultural Sciences, Urumqi, 830091, China

*Correspondence: Yuan Chen, Peng-bin Dong

Email addresses: chenyuan@gsau.edu.cn (Yuan Chen); dongpb@stumail.nwu.edu.cn (Pengbin Dong)

Table S1 Orthogonal test results of PGRs affect callus subculture

Test No.	A(NAA) $mg \cdot L^{-1}$	B(6-BA) $mg \cdot L^{-1}$	C(2,4-D) $mg \cdot L^{-1}$	D(KT) $mg \cdot L^{-1}$	Fresh weight(g)
I	1(0.2)	1(1.0)	1(0)	1(0)	1.199 ± 0.15^{d}
II	1(0.2)	2(1.5)	3(0.5)	2(0.2)	1.733 ± 0.15^{c}
III	1(0.2)	3(2.0)	2(0.2)	3(0.5)	1.837 ± 0.16^{c}
IV	2(0.5)	1(1.0)	3(0.5)	3(0.5)	1.812 ± 0.10^{c}
V	2(0.5)	2(1.5)	2(0.2)	1(0)	2.191 ± 0.27^{bc}
VI	2(0.5)	3(2.0)	1(0)	2(0.2)	2.884 ± 0.17^{a}
VII	3(1)	1(1.0)	2(0.2)	2(0.2)	1.944 ± 0.16^{bc}
VIII	3(1)	2(1.5)	1(0)	3(0.5)	2.457 ± 0.22^{ab}
IX	3(1)	3(2.0)	3(0.5)	1(0)	2.449±0.15ab

Values within each column are marked with different letters a indicate significant differences, as determined by Duncan's multiple range test ($p < 0.05$)

Table S2. ANOVA analysis and Range analysis on callus subculture obtained from the $L_9(3^4)$ orthogonal experiment.

Analytical method	A(6-BA) mg·L^{-1}	B(NAA) mg·L^{-1}	C(2,4-D) mg·L^{-1}	D(KT) mg·L^{-1}
ANOVA analysis				
Df	2	2	2	2
P value	0.0001**	0.0001**	0.0001**	0.1740
Range analysis				
k1	1.590	1.652	2.180	1.946
k2	2.300	2.127	1.991	2.187
k3	2.283	2.390	1.998	2.035
Rj	0.710	0.738	0.189	0.241
Best level	A_2	B_3	C_1	D_2

ki represents the average response of each factor at different levels where i denotes a level and k a factor. Rk =maxki -minki for each factor. NAA, naphthalene acetic acid; 2,4-D, 2,4-dichlorophenoxy acetic acid; 6-BA, 6–benzyladenine; KT, kinetin. 1 Value corresponds to the low level in the orthogonal design. 2 Value corresponds to the Middle level in the orthogonal design. 3 value corresponds to the high level in the orthogonal design. ** indicates extremely significant differences ($p < 0.01$).

Table S3. Factor load matrix.

Peaks	Principal component				
	PC1	PC2	PC3	PC4	PC5
1	0.741	0.078	0.457	0.342	0.118
2	0.767	0.047	0.368	0.490	0.151
3	0.817	0.177	0.342	0.318	0.135
4	0.512	0.474	0.573	0.293	0.049
5	0.041	0.897	0.028	0.163	0.298
6	0.418	0.702	0.226	0.117	0.318
7	0.283	0.550	0.539	0.339	0.087
8	0.447	0.015	0.506	0.676	0.205
9	0.577	0.629	0.309	0.106	0.195
10	0.502	0.376	0.125	0.064	0.716
11	0.796	0.144	0.415	0.364	0.105

Table S4. Characteristic values and contribution rates of principal components.

Principal component	Characteristic value	Contribution %	Cumulative contribution %
PC1	3.741	34.009	34.009
PC2	2.423	22.028	56.037
PC3	1.673	15.205	71.241
PC4	1.304	11.856	83.097
PC5	0.859	7.809	90.906
